# Protocatechuic acid inhibits LPS‐induced mastitis in mice through activating the pregnane X receptor

**DOI:** 10.1111/jcmm.17812

**Published:** 2023-06-16

**Authors:** Lihua Zhao, Lei Jin, Bin Yang

**Affiliations:** ^1^ Department of Breast Surgery China‐Japan Union Hospital of Jilin University Jilin China; ^2^ Department of Anesthesiology China‐Japan Union Hospital of Jilin University Jilin China

**Keywords:** LPS, mastitis, NF‐κB, pregnane X receptor, protocatechuic acid

## Abstract

Mastitis refers to the inflammation in the mammary gland caused by various reasons. Protocatechuic acid (PCA) exerts anti‐inflammatory effect. However, no studies have shown the protective role of PCA on mastitis. We investigated the protective effect of PCA on LPS‐induced mastitis in mice and elucidated its possible mechanism. LPS‐induced mastitis model was established by injection of LPS into the mammary gland. The pathology of mammary gland, MPO activity and inflammatory cytokine production were detected to evaluate the effects of PCA on mastitis. In vivo, PCA significantly attenuated LPS‐induced mammary pathological changes, MPO activity, TNF‐α and IL‐1β production. In vitro, the production of inflammatory cytokines TNF‐α and IL‐1β was significantly reduced by PCA. Furthermore, LPS‐induced NF‐κB activation was also inhibited by PCA. In addition, PCA was found to activate pregnane X receptor (PXR) transactivation and PCA dose‐dependently increased the expression of PXR downstream molecule CYP3A4. In addition, the inhibitory effect of PCA on inflammatory cytokine production was also reversed when PXR was knocked down. In conclusion, the protective effects of PCA on LPS‐induced mastitis in mice through regulating PXR.

## 1 INTRODUCTION

Mastitis is one of the biggest challenges in dairy farming and dairy products industry, which restricts the healthy development of dairy industry.^1^ Bacterial infection is the major cause of mastitis.^2^ LPS is the main component of the cell wall of *Gram‐negative* bacteria such as *Escherichia coli*.^3^ It has been known as the major pathogenic factor leading to mastitis.^4^ Stimulated by LPS, the release of inflammatory cytokines and oxidative mediators increased.^5^ Previous studies have shown that inflammation and oxidative stress play a huge role in the disease process of mastitis^6^, ^7^ and inhibition the inflammatory and oxidative responses had protective effects against mastitis.^8^ Antibiotics are the main agent for mastitis.^9^ However, with the long‐term and large‐scale abuse of antibiotics which caused the emergence of drug‐resistant strains.^10^ Therefore, it is of great significance to find safe and effective drugs to treat mastitis.

Pregnane X receptor (PXR) belongs to nuclear receptor superfamily.^11^ It can be activated by a variety of substances, both endogenous and exogenous.^12^ It is an important regulatory factor for drug metabolism detoxification.^13^ PXR not only plays a role in drug metabolism and toxicity, but also plays a regulatory role in cancer occurrence, metastasis, apoptosis, drug resistance and prognosis.^14^, ^15^ Recently, PXR has been known to regulate the inflammatory response.^16^ Previous studies demonstrated that PXR agonists could prevent inflammation and NF‐κB signalling pathway.^17^, ^18^

Protocatechuic acid (PCA), an active plant phenolic acid, exhibits anti‐inflammatory and oxidative effects.^19^ PCA was found to attenuate inflammation in LPS‐challenged piglets.^20^ Previous studies showed that in the LPS‐induced mouse acute lung injury model, PCA can inhibit the occurrence and development of the disease.^21^ In BV2 cells cultured in vitro, PCA also played a similar role, which could inhibit the production of inflammatory cytokines in the cells.^22^ A previous study showed that PCA inhibited LPS‐induced inflammation in human gingival fibroblasts.^23^ However, the protective effects of PCA on mastitis and the possible mechanism have not been clarified. In the present study, we investigated the protective roles of PCA on mastitis in mice.

## 2 MATERIALS AND METHODS

### 2.1 Materials

LPS (Escherichia coli 055:B5) was purchased from Sigma (St. Louis, MO, USA). Mouse TNF‐α and IL‐1β ELISA kits were obtained from R&D systems (Minneapolis, MN, USA). PCA (purity>98%) was purchased from Shanghai Pure One Biotechnology (Shanghai, China). PXR and NF‐κB specific antibodies were obtained from CST (Beverly, USA).

### 2.2 Mastitis model and treatment

Sixty lactating female mice 5–7 days after delivery were used in this study. To establish an LPS‐induced mastitis model, LPS (1 μg/μL) was injected into the udder canals of L4 (left) and R4 (right), and tissue samples were collected 24 h later. 20 mg PCA was dissolved in 200 μL DMSO and further dissolved in PBS for the indicated concentration. PCA (10, 20 and 30 mg/kg) were given intraperitoneal injection. The doses of PCA were based on previous studies.^24^ Experimental protocol was approved by the Ethical Committee of Jilin University.

### 2.3 H&E staining

The mammary tissue was fixed with 100 mL/L formaldehyde solution, embedded in paraffin, sectioned and stained with H&E for microscopic observation of pathological changes of mammary tissue.

### 2.4 ELISA and MPO assays

Inflammatory cytokines TNF‐α and IL‐1β in mammary tissues and cell culture supernatant was detected by the ELISA Kit. MPO level in mammary tissues was detected by MPO detection kit.

### 2.5 Cell culture and treatment

EpH4‐Ev cells (a mouse mammary epithelial cell) were cultured in DMEM containing 10% FBS at 37°C. 20 mg PCA was dissolved in 200 μL DMSO and further dissolved in DMEM medium to the concentration of 5, 10, 20 μM. After cells were treated with PCA (5, 10, 20 μM) for 1 h, LPS was added for 24 h. The concentration of PCA (5, 10, 20 μM) in vitro used in this study was based on previous studies.^22^, ^23^ Cytokine production and the NF‐κB signalling pathway were examined by ELISA and Western blot analysis.

### 2.6 PXR knockdown experiment

EpH4‐Ev cells were plated in six well plates and cultured to 80% confluence. Then, the cells were transfected with PXR siRNA (100 nM) or control siRNA (100 nM) using the Lipofectamine 2000 transfection reagent (Thermo, USA). 24 h after transfection, the cells were treated with PCA (20 μM) or rifampicin (10 μM), followed by the treatment of LPS 24 h. rifampicin was used as a positive control. Finally, TNF‐α and IL‐1β production were measured. The inhibition of siRNA on PXR expression was detected by western blot analysis.

### 2.7 PXR transactivation assay

PXR transactivation assay was detected as described previous.^25^ In brief, HEK293 cells were seeded in 24 well plate (each well contains 1 × 10^5^ cells). 24 h later, the cells were transiently transfected 200 ng pSG5‐hPXR (provided by Dr Li, Jilin Agricultural University) and 5 ng pcDNA3‐Rluc (Promega, USA) as an internal control for normalization. 6 h later, the cells were treated with PCA (5, 10, 20 μM) for 48 h. Then, the cells were collected and the luciferase activity was detected.

### 2.8 Western blot assay

Total proteins of the cells were extracted using protein extraction kit (Thermo, USA). Samples of proteins (40 μg) were loaded on 12% SDS‐PAGE. After electrophoretic separation of the proteins, the proteins on the gel were transferred to a solid support under the action of an electric field and blocked with 5% BSA. Immunodetection is performed using a chemiluminescent substrate after binding the membrane to the primary antibodies (1:1000) and secondary antibodies (1:5000).

### 2.9 Statistical analysis

Data were presented as mean ± SEM, and data analysis was performed using SP SS18.0 statistical software. Differences between groups were analysed by one‐way analysis of variance, and the ratio between the two groups was measured by Newman‐Kuels *q* test, with *p* < 0.05 is statistically significant.

## 3 RESULTS

### 3.1 Effects of PCA on LPS‐induced mammary pathological damage

**FIGURE 1:**
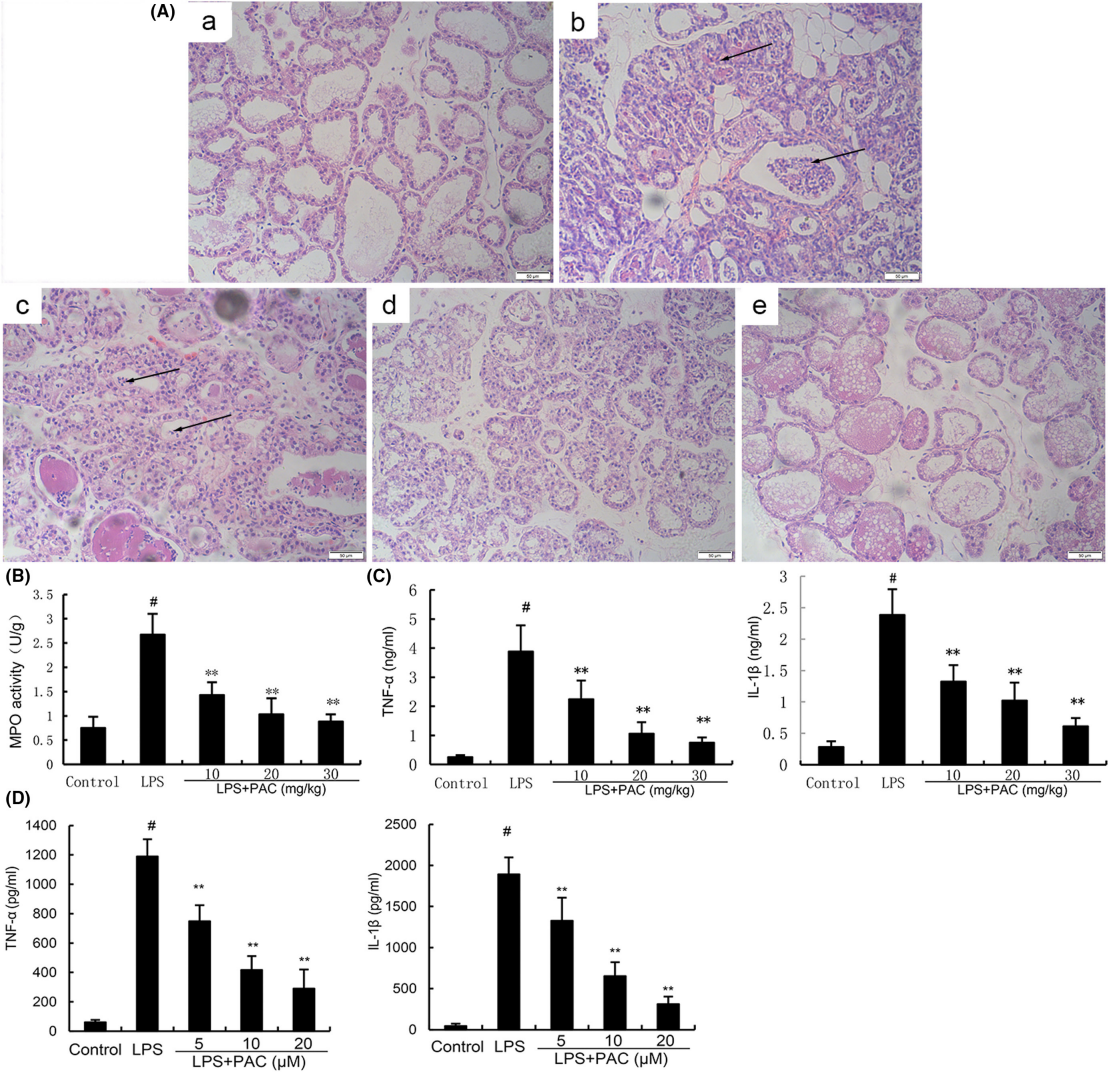
(A) Effects of PCA on LPS‐induced mammary histopathological changes. Histopathologic sections of mammary tissues (H&E, × 100). (a) Mammary tissues from control, (b) LPS, (c) LPS + PCA (10 mg/kg), (d) LPS + PCA (20 mg/kg), (e) LPS + PCA (30 mg/kg). (B) Effect of PCA on MPO activity in mammary gland tissues induced by LPS. (C) Effect of PCA on cytokine production in mammary gland tissues induced by LPS. (D) Effects of PCA on LPS‐induced TNF‐α and IL‐1β production in EpH4‐Ev cells. The data of this study are presented as mean ± SEM of three parallel measurements. The values presented are means ± SEM. #*p* < 0.01 is significantly different from the control group; ** *p* < 0.01 are significantly different from the LPS group.

To investigate the protective roles of PCA on LPS‐induced mastitis, mammary pathological injury was detected in this study by H&E staining. The control group did not show obvious pathological histological changes (Figure 1A). As shown in Figure 1B, LPS led to severe mammary pathological injury.^26^ As the arrows shown, LPS stimulation caused hyperemic edema in the acinar cavity, and acinar cavity was infiltrated with a large number of inflammatory cells (Figure 1B). However, LPS‐induced mammary pathological injury was alleviated by PCA treatment (Figure 1C, D, E).

### 3.2 PCA attenuated LPS‐induced MPO activity

MPO activity is a biomarker of inflammatory cell infiltration.^27^ In this study, we detected the effects of PCA on LPS‐induced inflammatory cell infiltration by measuring MPO activity in mammary tissues. MPO activity in mammary tissues of the LPS group increased markedly (0.75 ± 0.23 vs. 2.67 ± 0.43). PCA (10, 20, and 30 mg/kg) dose‐dependently attenuated LPS‐induced increase in MPO activity (1.43 ± 0.26, 1.03 ± 0.33, 0.88 ± 0.15 vs. 2.67 ± 0.43), which supported PCA inhibited neutrophil infiltration (Figure 1B).

### 3.3 Effects of PCA on LPS‐induced TNF‐α and IL‐1β production

TNF‐α and IL‐1β are important inflammatory cytokines that play critical role in the development of mastitis. We detected the production of TNF‐α and IL‐1β in mammary tissues to assess the anti‐inflammatory role of PCA. TNF‐α (0.25 ± 0.07 vs. 3.88 ± 0.89) and IL‐1β (0.28 ± 0.09 vs. 2.38 ± 0.41) in mammary tissues of the LPS group were higher than the control group. PCA (10, 20, and 30 mg/kg) dose‐dependently alleviated LPS‐induced TNF‐α (2.24 ± 0.64, 1.05 ± 0.39, 0.75 ± 0.18 vs. 3.88 ± 0.89) and IL‐1β (1.32 ± 0.26, 1.02 ± 0.28, 0.61 ± 0.13 vs. 2.38 ± 0.41) production, which supported the anti‐inflammatory role of PCA (Figure 1C). In vitro, PCA (5, 10, 20 μM) significantly inhibited LPS‐induced TNF‐α (748.73 ± 108.12, 416.96 ± 93.78, 289.42 ± 131.36 vs. 1189.81 ± 116.4) and IL‐1β (1326.02 ± 282.24, 651.25 ± 171.16, 312.41 ± 90.87 vs. 1892.81 ± 282.24) production (Figure 1D).

### 3.4 Effects of PCA on LPS‐induced NF‐κB activation

**FIGURE 2:**
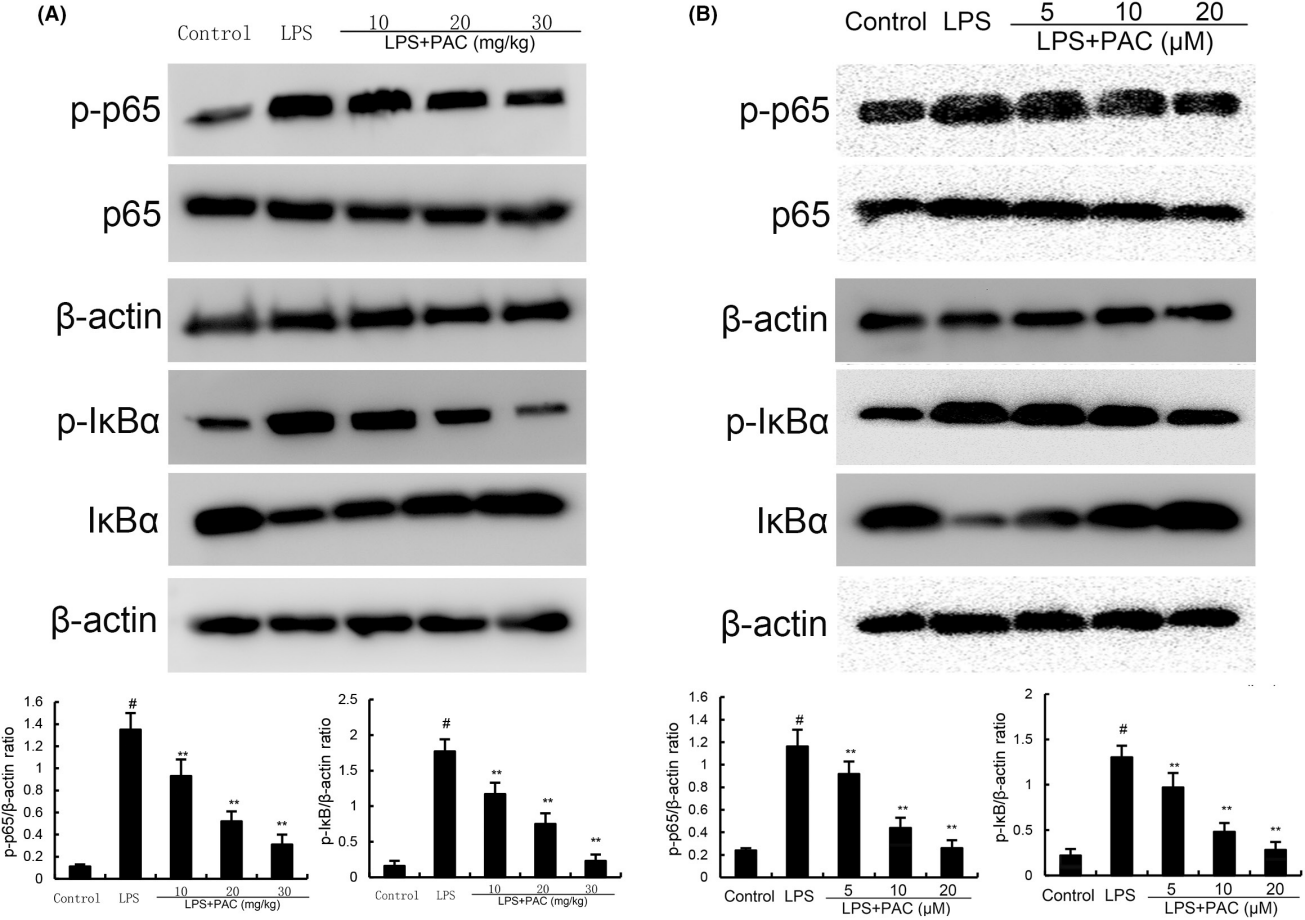
(A) Effects of PCA on LPS‐induced NF‐κB activation in mammary tissues. The data are presented as mean ± SEM of three parallel measurements (*n* = 6). (B) Effects of PCA on LPS‐induced NF‐κB activation in EpH4‐Ev cells. The data of this study are presented as mean ± SEM of three parallel measurements. *p*# < 0.01 versus control group, p** < 0.01 versus LPS group.

NF‐κB controls a variety of immune‐related genes, such as TNF‐α and IL‐1β. To clarify the mechanism responsible for the anti‐inflammatory effects of PCA, we measured NF‐κB activation by western blot analysis. The levels of NF‐κB p65 and IκBα phosphorylation of the LPS group were higher than the control group. PCA markedly alleviated LPS‐induced increases in NF‐κB p65 and IκBα phosphorylation (Figure 2A,B).

### 3.5 Effects of PCA on PXR activation

**FIGURE 3:**
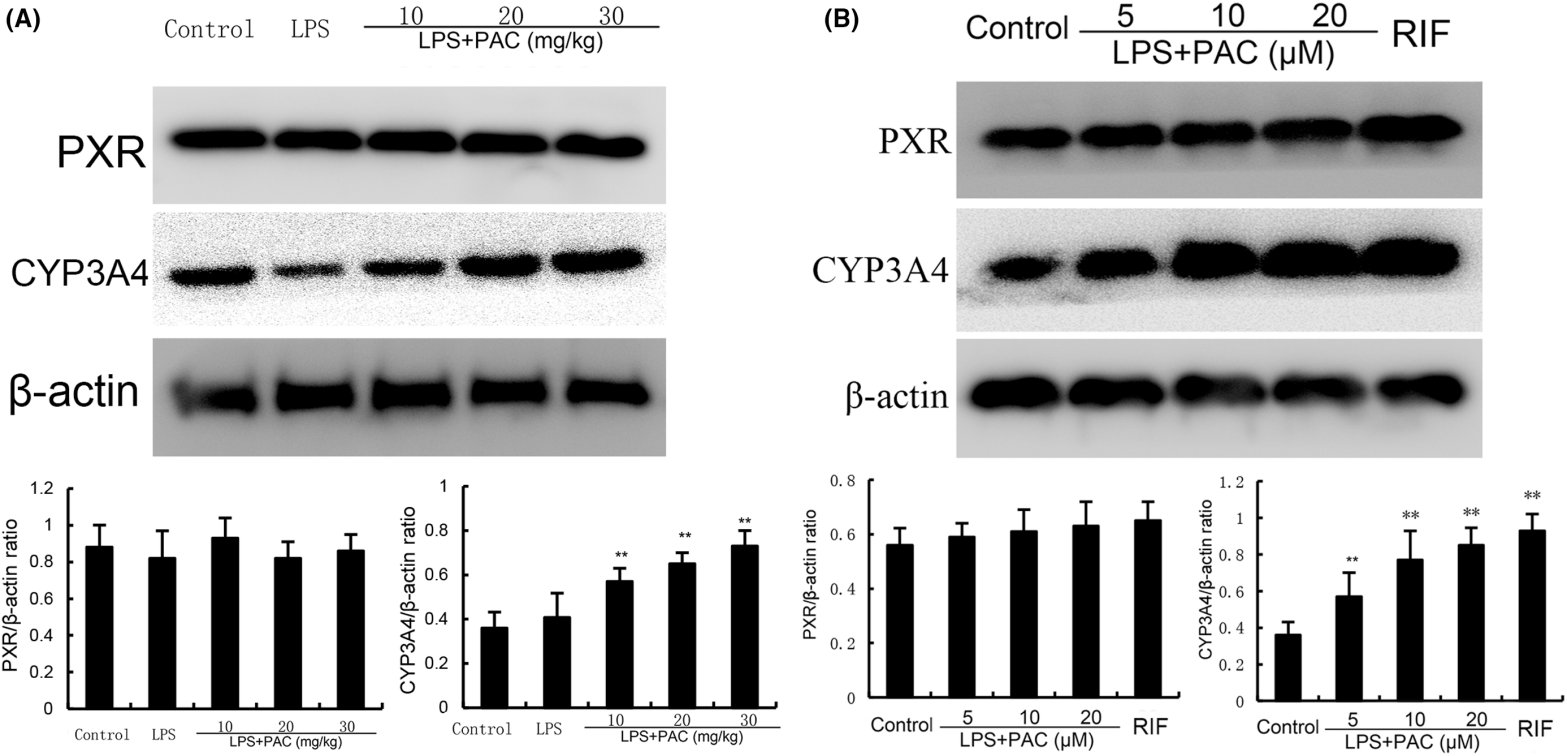
(A) Effects of PCA on PXR and CYP3A4 expression in mammary tissues. The data are presented as mean ± SEM of three parallel measurements (*n* = 6). *p*# < 0.01 versuss control group, p** < 0.01 versus LPS group. (B) Effects of PCA on PXR and CYP3A4 expression in EpH4‐Ev cells. RIF was used as a positive control. The data of this study are presented as mean ± SEM of three parallel measurements. p** < 0.01 versus control group.

**FIGURE 4:**
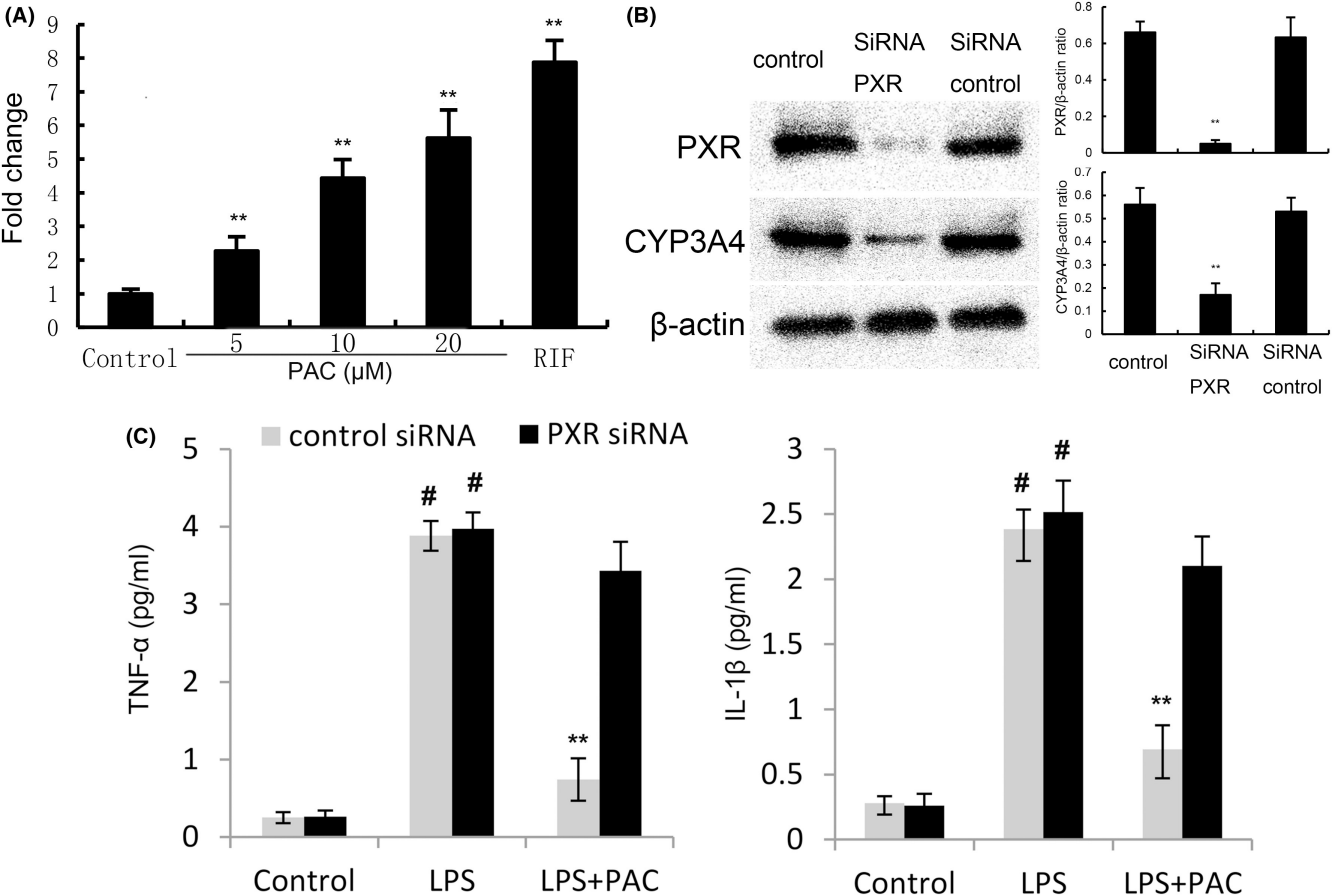
(A) Activation of PXR transactivity by PCA in HEK293 cells. The data of this study are presented as mean ± SEM of three parallel measurements. p** < 0.01 versus control group. (B) Effects of PXR siRNA on RXR and CYP3A4 expression in EpH4‐Ev cells. (C) FOR inhibits LPS‐induced inflammation via activating PXR in EpH4‐Ev cells. The data of this study are presented as mean ± SEM of three parallel measurements. *p*# < 0.01 versus. control group, p** < 0.01 versus. LPS group.

PXR, as an important nuclear receptor transcription factor, has the ability to regulate the activation of NF‐κB. To further clarify the anti‐inflammatory mechanism of PCA, PXR activation was detect. To test the effects of PCA on PXR activation, the expression of PXR and its downstream molecule CYP3A4 were detected. PCA did not affect the expression of PXR but dose‐dependently increased the expression of PXR downstream CYP3A4 both in vivo (0.57 ± 0.06, 0.65 ± 0.05, 0.73 ± 0.07 vs. 0.47 ± 0.11) and in vitro (0.55 ± 0.06, 0.78 ± 0.08, 0.83 ± 0.04 vs. 0.38 ± 0.04) (Figure 3A, B). Furthermore, luciferase assay showed that PCA could activate PXR (Figure 4A). These results suggested that PCA had a significant activation effect of PXR.

### 3.6 PCA inhibits LPS‐induced inflammation through activating PXR

To investigate whether PCA exhibited anti‐inflammatory effects through activating PXR, PXR was knockdown by siRNA (Figure 4B). Furthermore, the inhibition of PCA on LPS‐induced TNF‐α and IL‐1β production were reversed when PXR was knockdown (Figure 4C).

## 4 DISCUSSION

Natural herbal medicines and its compound have been known as valuable sources for drug development.^28^ PCA exhibits anti‐inflammatory and oxidative roles. Previous studies demonstrated PCA could exert relatively good anti‐inflammatory roles in disease models.^29^ We showed PCA protected mice against LPS‐induced mastitis through enhancing autophagy via activating the PXR.

Neutrophils play a huge role in the development of mastitis. It can significantly defend against the invasion of pathogens.^30^ Numerous studies have shown that the number of neutrophils increases dramatically during the disease process of mastitis.^31^ These neutrophils could release inflammatory and oxidative mediators.^32^ In this study, we observed that elevated TNF‐α and IL‐1β in the mammary tissues of mice in the LPS group. Meanwhile, we found PCA significantly inhibited these inflammatory cytokine productions. Furthermore, LPS‐induced neutrophils infiltration in mammary tissues was attenuated by PCA. We found PCA significantly prevented LPS‐induced inflammation.

LPS‐induced inflammatory cytokine production was mainly mediated by NF‐κB.^33^ This cascade leads to the gene transcription of TNF‐α and IL‐1β.^34^ In previous studies, many herbal medicines protected mice against mastitis by inhibiting NF‐κB activation.^35^, ^36^ In this study, PCA significantly suppressed LPS‐induced NF‐κB activation. PXR, as an important nuclear receptor transcription factor, widely participates in various physiological activities, including drug metabolism and drug resistance.^37^ Recently, it has been known that activation of PXR could regulate inflammatory response.^38^ PXR agonists have been reported to inhibit LPS‐induced NF‐κB activation and inflammatory responses.^39^ CYP3A4 is an enzyme involved in the metabolism of numerous drugs which encodes one of the most important Phase I drug‐metabolizing enzymes in humans. CYPA3A4 is one of the PXR target genes. The anti‐inflammatory effects of many Chinese herbal compounds are exerted by activating PXR‐mediated CYPA3A4 activation.^40^, ^41^ Our data showed that PCA did not affect the expression of PXR in mammary gland tissues. PCA dose‐dependently increased the expression of PXR downstream CYP3A4 in mammary tissues. In most studies, the expression of PXR was not changed when PXR was activated and PXR activation was usually tested by detecting PXR transactivation and PXR downstream CYP3A4 expression. Our results showed that PCA did not affect the expression of PXR but increased the expression of PXR downstream CYP3A4. Furthermore, luciferase assay showed that PCA could activate PXR. These results suggested that PCA had a significant activation effect on PXR. Furthermore, the inhibition of PCA on LPS‐induced inflammatory cytokine production was inhibited when PXR was knockdown. After PXR knockdown, PCA no longer exerted an inhibitory effect on inflammatory cytokine production. These results suggested that PXR‐mediated activation of CYP3A4 could be one of the mechanisms of PAC.

In conclusion, we suggest that PCA exerts a considerable protective role in LPS‐induced mastitis. The protective mechanism of PCA on LPS‐induced mastitis in mice may be related to the regulation of PXR.

## AUTHOR CONTRIBUTIONS

**Lihua Zhao:** Data curation (equal); formal analysis (equal); investigation (equal); methodology (equal); software (equal); writing – original draft (equal). **Lei Jin:** Data curation (equal); investigation (equal); methodology (equal); supervision (equal); validation (equal). **Bin Yang:** Conceptualization (equal); data curation (equal); project administration (equal); supervision (equal); validation (equal); writing – review and editing (equal).

## CONFLICT OF INTEREST STATEMENT

The authors have no relevant financial or non‐financial interests to disclose.

## CONSENT FOR PUBLICATION

All authors agree to publish in Journal of Cellular and Molecular Medicine.

## Data Availability

The data that support the findings of this study are available from the corresponding author upon reasonable request.
